# The Mediating Role of Non-reactivity to Mindfulness Training and Cognitive Flexibility: A Randomized Controlled Trial

**DOI:** 10.3389/fpsyg.2020.01053

**Published:** 2020-06-26

**Authors:** Yingmin Zou, Ping Li, Stefan G. Hofmann, Xinghua Liu

**Affiliations:** ^1^Beijing Key Laboratory of Behavior and Mental Health, School of Psychological and Cognitive Sciences, Peking University, Beijing, China; ^2^Department of Psychological and Brain Sciences, Boston University, Boston, MA, United States

**Keywords:** Mindfulness Based Stress Reduction, cognitive flexibility, non-reactivity, mediation, mechanism

## Abstract

Mindfulness training has been shown to have a beneficial effect on cognitive flexibility. However, little is known about the mediators that produce this effect. Cross-sectional studies show that there might be a link between Non-judgment, Non-reactivity and cognitive flexibility. Longitudinal studies examining whether Non-judgment or Non-reactivity mediate the effectiveness of mindfulness training on improving cognitive flexibility are lacking. The present study aims to test the effect of mindfulness training on increasing cognitive flexibility and to test whether this effect is mediated by Non-judgment or Non-reactivity. We conducted a single-blind randomized controlled trial in 54 nonclinical high-stress participants between October 2018 and January 2019. Participants were randomly assigned to a Mindfulness Based Stress Reduction (MBSR) group or a waitlist control group. The experimenters were blind to the group assignment of participants. The MBSR group received 8-weekly sessions (2.5-h per week) and a one-day retreat (6-h), and was required to accomplish a 45-min daily formal practice during the intervention. The waitlist control group did not receive any intervention during the waiting period and received a 2-day (6-h per day) mindfulness training after the post-intervention. The primary outcome was self-report cognitive flexibility and perceived stress administered before and after MBSR. The secondary outcome was self-report mindfulness skills (including Non-reactivity and Non-judgment) measured at pre-treatment, Week 3, Week 6, and post-intervention. For cognitive flexibility, mixed-model repeated-measure ANOVA results showed that there were significant main effects of Time, Group and a significant interaction of Time by Group. Follow-up ANOVA indicated that the MBSR group was associated with greater improvements in cognitive flexibility than the waitlist. Path analysis results showed that the effect of the treatment on cognitive flexibility at post-treatment was fully mediated by Non-reactivity at Week 6. The mediation effects of Non-reactivity at Week 3, and Non-judgment at Week 3 and Week 6 were not significant. Our findings support the efficacy of MBSR on improving cognitive flexibility. Non-reactivity is an important element of the effectiveness of MBSR training on cognitive flexibility.

## Introduction

Mindfulness has been defined as attention or awareness to present-moment experiences with acceptance (Baer, 2003; Kabat-Zinn, 2003; Bishop et al., 2004). Importantly, mindfulness is an innate capacity of humans. At the same time, it can be fostered and deepened by mindfulness based interventions (MBIs) (Lindsay and Creswell, 2017), such as the Mindfulness Based Stress Reduction Program (MBSR) (Kabat-Zinn, 1990) and Mindfulness Based Cognitive Therapy (MBCT) (Segal et al., 2012). MBI alleviates psychological distress (e.g., stress, anxiety, mood symptoms) with medium effect sizes compared to waitlist controls (Hedge’s gs = 0.41−0.53), and active treatment controls (Hedge’s gs = 0.33−0.5) (Hofmann et al., 2010; Khoury et al., 2013). Additionally, preliminary evidence supports that MBI enhances cognitive abilities (e.g., cognitive flexibility, attention, and executive functioning), which might affect social functioning (Lutz et al., 2015; Li et al., 2018; Wielgosz et al., 2019). Some studies suggest that cognitive flexibility promotes effective management of stressful life events, and is associated with good mental health (Kashdan and Rottenberg, 2010; Logue and Gould, 2014).

Cognitive flexibility is conceptualized as the ability to flexibly and adaptively respond to the environments, as opposed to the rigid or automatic thinking style, triggered by prior experience (Hayes et al., 1999; Shapiro et al., 2006; Dennis and Vander Wal, 2010). Lack of cognitive flexibility, or cognitive rigidity, is an important vulnerability for the development and maintenance of psychological distress (Morris and Mansell, 2018). When confronted with difficult life situations, individuals with a rigid thinking style tend to perceive the situation as unchangeable and uncontrollable and tend to engage in rumination, leading to distress in the long term. If individuals see only one solution to a difficult life situation, they might perceive themselves as incapable of problem solving. That might interfere with their long-term goals, which might further increase emotional distress. As part of cognitive behavioral therapy (CBT), psychological distress is alleviated by targeting maladaptive and rigid automatic cognitions with more adaptive cognitions (Derubeis et al., 1991; Chambless and Gillis, 1993). In this context, Dennis and Vander Wal (2010) developed a self-report instrument, the Cognitive Flexibility Inventory (CFI), to measure cognitive flexibility. The CFI consists of two factors, namely the Control factor and the Alternative factor. Items on the Control factor measure the degree to which individuals perceive the difficult life situation as controllable. Items on the Alternative factor denote the extent to which individuals perceive multiple explanations and solutions to the difficult life situation. It seems likely that individuals with flexible and adaptive cognitions experience less psychological distress than those with rigid thinking styles. In fact, a greater level of perceived control has been shown to be associated with higher tendency to adapt coping strategies to different stressful life situations (Cheng and Cheung, 2005). Furthermore, individuals with higher levels of perceived control tend to accommodate with life stressors including economic difficulties, unemployment and care-given burdens (Zautra et al., 2012). Less dichotomous thinking (e.g., If I fail at my work, then I am a failure as a person), was indicative of alleviated perceived stress (Otto et al., 1997; Ford and Shook, 2018). Meanwhile, it is evident that an increase in perceived problem solving capability predicted less perceived stress longitudinally (Otto et al., 1997), suggesting that flexible cognitions contribute to successful management of life event stress.

Mindfulness has long been proposed to be associated with cognitive flexibility. Some researchers have proposed that cognitive flexibility is a component of mindfulness (Bishop et al., 2004; Chanowitz and Langer, 1981; Feldman et al., 2007; Frewen et al., 2008; Moore and Malinowski, 2009). For example, Chanowitz and Langer (1981) defined mindfulness as a consciousness state or a mode of cognitive functions that would allow individuals to get actively involved in reframing the environment. This, in turn, might enable individuals to draw voluntary attention on contextual cues, leading to flexible and adaptive cognitions or behaviors. Bishop et al. (2004) suggested that mindfulness is operationally defined as the self-regulation of attention and orientation to the experience. Being cognitive flexible is considered an important component of self-regulation of attention. However, relatively little is known about the role of cognitive flexibility in mindfulness (Kee and Wang, 2008). Moore (2013) has shown that cognitive flexibility is positively associated with mindfulness and contributed to flow experiences when controlled for mindfulness, suggesting that cognitive flexibility and mindfulness are independent but correlated constructs. Similar to cognitive flexibility, mindfulness was found to be associated with lower levels of perceived stress (Senders et al., 2014; Gustafsson et al., 2015). Shapiro et al. (2006) proposed that mindfulness trainings might facilitate awareness of one’s habitual reactions and enable individuals to see the present situation as it is and respond adaptively and flexibly. So far, only one study has shown that MBI improves self-report cognitive flexibility. Shapero et al. (2018) found that for depressed individuals, participants receiving MBCT training reported higher levels of cognitive flexibility than a waitlist group. In sum, mindfulness is positively associated with cognitive flexibility and both of them are associated with lower emotional distress. On top of that, emerging evidence suggests that MBIs might be effective for improving cognitive flexibility.

Although mindfulness has been shown to cultivate adaptive and flexible responses, the mechanism producing this effect requires further exploration. Theoretical models have provided fundamental insights for the underlying mechanism. The mindfulness stress-buffering theory (Creswell and Lindsay, 2014) proposes that acceptance is the main ingredient of mindfulness training on adaptive responses for stress. Acceptance is often defined as openness toward emotion and experience (Campbell-Sills et al., 2006). The ability to accept stressors buffers the habitual appraisals and responses, which in turn facilitates new appraisals and coping strategies. Studies have shown that the association between trait mindfulness with peace of mind was mediated by acceptance (Xu et al., 2015), and the positive link between mindfulness and subjective well-being was significantly mediated by self-acceptance only (Xu et al., 2016). Moreover, accepting pain increased pain endurance and tolerance after training than simply paying attention to the pain without accepting it (Wang et al., 2019).

It has been suggested that accepting an experience might be cultivated by approaches that encourage individuals to fully experience their bodily sensations, emotions, and thoughts without changing or avoiding them (Hayes et al., 1999). However, little is known about the specific mindfulness-based approach that fosters this acceptance attitude. Lindsay and Creswell (2017) proposed that mindfulness training might foster acceptance through non-judgmental (without judging them as good or bad) and non-reactive (without reacting to change them) attitudes toward internal and external experiences. Mindfulness practices emphasize Non-judgment by allowing for any experience arising in our mind, without evaluating them as good or bad. Thus, this process may be presumed to shift habitual stress appraisal sets. Non-reactivity is accomplished through allowing experiences to come and go without reacting in an effort to change them. Non-reactivity is important in explaining the reduction of mood symptoms gained by mindfulness training. After a 3-month training, Non-reactivity predicted more reduction of mood symptoms in a present awareness mindfulness training group as compared to a progressive muscle relaxation training group (Gao et al., 2018). Theoretically, Non-reactivity may buffer the stress reactivity, which in turn would permit the generation of new responses, thus increasing cognitive flexibility (Kuyken et al., 2010; Dajani and Uddin, 2015; Van Der Velden and Roepstorff, 2015). Baer et al. (2012) reported that Non-judgment and Non-reactivity both showed significant improvements from baseline to Week 3 and Week 6 of a mindfulness intervention. Therefore, Week 3 and 6 might be two critical time points when changes in Non-judgment and Non-reactivity during mindfulness training occurs. Although theoretical models make reasonable assumptions, empirical evidence is relatively lacking. Currently, the pathways linking mindfulness training, Non-judgment/Non-reactivity and cognitive flexibility are poorly understood.

The present study aims to examine the effect of MBSR on cognitive flexibility and the mediating role of Non-judgment and Non-reactivity among them. Based on the previous studies, we hypothesized that: (1) Compared to a waitlist control group, the MBSR group will show elevated cognitive flexibility scores at post-intervention; (2) Non-reactivity scores during the intervention will mediate the treatment-induced changes in cognitive flexibility at the post-treatment assessment point; and (3) Non-judgment scores during the intervention will significantly mediate the relationship between intervention group and cognitive flexibility scores at post-intervention.

## Materials and Methods

### Participants

One hundred and two participants were recruited via social media advertisement. The inclusion criteria were: (a) a score on the Chinese Perceived Stress Scales (CPSS; Yang and Huang, 2003) ≥26; (b) having no prior experience with the 8-week MBSR or MBCT protocol; (c) a practice frequency of yoga, meditation, or Tai chi less than 20 min per week in the past six months; (d) absence of severe or unstable physical illness that would prevent one from attending trainings; and (e) a commitment to the group setting (e.g., randomization, no schedule conflicts, no attendance to other MBI or experiments during training). Participants were excluded if they met the DSM-IV-TR criteria (American Psychiatric Association [APA], 1994) for any diagnosis in the past six months. They were excluded if they had any self-injury or suicidal risks, aggression or destructive behaviors. The trial was conducted between October 2018 and January 2019, at the Peking University, Beijing, China.

### Procedure

Participants were invited to complete a survey attached to the advertisement. The survey included questions about personal experiences and information (e.g., the prior experience about MBSR or MBCT; the practice frequency of yoga, meditation, or Tai chi; the physical condition), and the CPSS. A study staff member subsequently telephoned to confirm the participant was able to commit to the group setting. Meanwhile, they were invited to attend the Structured Clinical Interview for DSM-IV-TR (First et al., 2002) conducted by a psychiatrist. The CONSORT checklist (Schulz et al., 2010) of this clinical trial is displayed in Supplementary Material.

After eligibility assessment, 54 participants were included. To match the gender and age between the MBSR group and waitlist group, a research assistant used a stratified random method to allocate participants. First, the age range was calculated. Then, the potential number of strata was assigned an integer that could be divided by the age range. The optimal number of strata was reached when the gender ratio within each strata became approximately 1:1. In our study, eight was chosen as the final strata number. The randomization was carried out within each strata. The random number sequence from 1 to 100 was generated by Excel. Each participant was allocated to a random number. This random number was divided by 2. If the remainder was 0, the corresponding participant was allocated to the MBSR group. If the remainder was 1, the participant was allocated to the waitlist group.

**FIGURE 1:**
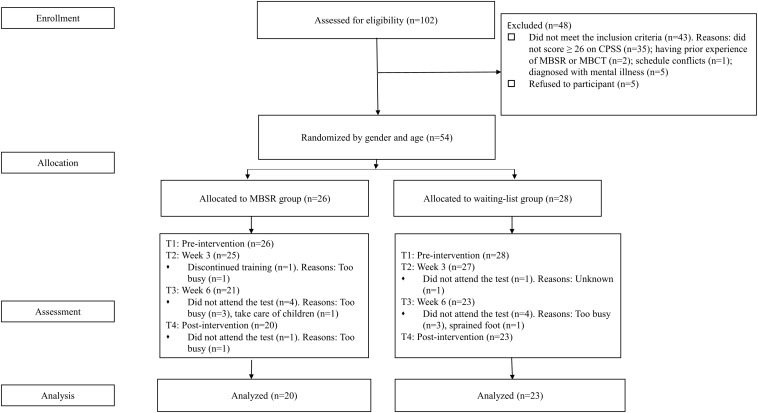
Flow chart of study procedure.

Twenty-six participants were allocated to the MBSR group, and 28 were allocated to the waitlist group (allocation ratio = 13:14). Group assignment was done by the research assistant. The participants would not be informed of their assignment until they completed the pre-test. Before the pre-test, all participants gave their informed consent. The intervention started on November 2018 and ended on January 2019. The MBSR group received the 8-week (2.5-h per week) sessions and a one-day retreat (a weekend between Week 6 and Week 7), led by two instructors adhering to the MBSR developed by Kabat-Zinn (1990). Meanwhile, participants were asked to practice guided meditation for 45 min daily. The waitlist group was not offered any kind of intervention during the waiting period, but they had access to a 2-day mindfulness training after the post-intervention. Participants were asked to complete the self-report questionnaires at 4 time points based on the timeline of MBSR group: pre-treatment (pre randomization, T1), Week 3 test (T2), Week 6 test (T3), and post-treatment (the week following week 8, T4) (for the flowchart of the participants, see Figure 1). The questionnaires were delivered to the participants via an online link before the session. Participants had 40 min for completing the measures. Within the training period, the participants and the instructors were not blind to the group assignment, only the experimenters were blind. Participants who finished all the tests were thanked and received 100 RMB as compensation. Our study protocol was approved by the Association for Ethics and Human and Animal Protection in School of Psychological and Cognitive Sciences, Peking University (No. 2018-10-02).

### Measures

The Chinese version (Deng et al., 2011) of the Five Facet Mindfulness Questionnaire (FFMQ; Baer et al., 2008) was used to assess the tendency to be mindful. The FFMQ consists of 39 items with a 5-point Likert rating scale (1 = never or very rarely true, 5 = very often or always true). Its five-factor construct is reliable and valid in English and Chinese settings, which refers to Observing (e.g., “I notice the smells and aromas of things”), Describing (e.g., “I am good at finding words to describe my feelings”), Acting with awareness (e.g., “I find myself doing things without paying attention,” reverse coding), Non-reactivity to inner experiences (e.g., “I perceive my feelings and emotions without having to react to them”), and Non-judging of inner experiences (e.g., “I think some of my emotions are bad or inappropriate and I should not feel them,” reverse coding). A higher score indicates that one is more mindful in everyday life. If the mediating role of Non-judgment and Non-reactivity is to be verified, their changes have to emerge prior to the changes of outcome variables (Kazdin, 2007). Thus, the FFMQ was evaluated during the MBSR intervention in addition to the pre- and post-treatment. In the present study, the Cronbach’s αs of the FFMQ, and the five subscales across 4 time points ranged from 0.84 to 0.93.

The Chinese version (Wang et al., 2016) of the CFI (Dennis and Vander Wal, 2010) was utilized to assess the ability to generate alternative explanations and solutions to difficult situations. The CFI is comprised of 20 items utilizing a 1–5 point Likert rating scale (1 = never, 5 = always). It assesses two aspects of cognitive flexibility: the proneness to perceive difficulties as controllable (e.g., “When I encounter difficult situations, I feel like I am losing control,” “I am capable of overcoming the difficulties in life that I face”), and the capability to generate multiple explanations and solutions when confronted with life events and difficulties (e.g., “I consider multiple options before making a decision,” “I like to look at difficult situations from many different angles”). The original CFI has good internal consistency (Cronbach’s αs = 0.84−0.92), 7-weeks test-retest reliability (*r* = 0.81) and construct validity for clinical and non-clinical samples (Dennis and Vander Wal, 2010). The Chinese version showed good internal consistency (Cronbach’s αs = 0.81) and revealed a two-factor structure, consistent with the original scale. Higher scores indicate more flexibility in cognitive appraisal and problem solving when encountering difficult situations. To examine the efficacy of MBSR on cognitive flexibility, the CFI was administered at pre- and post-treatment. The Standardized Response Mean (SRM) (the mean difference between pre- and post-treatment divided by the standard deviation of the difference), was 0.93, indicating large sensitivity to change (Cohen, 1992). In the present study, the Cronbach’s αs of CFI were at 0.75 pre-intervention, and 0.91 at post-intervention.

The CPSS (Cohen et al., 1983; Yang and Huang, 2003) was administered to evaluate the degree to which individuals perceived their situations as uncontrollable, unpredictable, and unresolvable in the past month. The CPSS includes 14 items (e.g., “I feel intense and stressful,” “I feel that the problem is constantly accumulating and cannot be solved”) with a 5-ponit Likert rating scale (0 = never, 4 = always). It exhibits great internal consistency and construct validity in English and Chinese settings (Cohen et al., 1983; Yang and Huang, 2003). A higher score indicates a higher level of perceived stress. The CPSS was conducted at pre and post-treatment in order to capture the stress reduction effect of MBSR. In the present study, the Cronbach’s αs of CPSS were 0.72 at pre-treatment, and 0.86 at post-treatment.

### Data Analyses

First, we utilized G^∗^power 3.1 (Faul et al., 2007) to compute the required sample size. Based on a previous study (Shapero et al., 2018), we considered a between-group effect size (η^2^) of 0.24 regarding the mindfulness training effect on cognitive flexibility. To obtain power of 0.8 with two measurement points, the total sample size of 50 would be sufficient to detect a significant Group × Time interactions by repeated-measure ANOVAs at *p* < 0.05.

Second, we conducted the missing value analysis with the Statistical Package for Social Sciences (version 17.0 for Windows; SPSS Inc., Chicago, IL, United States). All data were analyzed using multivariate intention-to-treat analyses. For FFMQ, CFI, and CPSS, Little’s MCAR (Missing Completely At Random) tests showed that data were missed at random (*p*s > 0.05). We used the expectation-maximization method suggested by Newgard and Lewis (2015) to impute the missing data. We compared group differences in age, gender, educational years, FFMQ, CFI, and CPSS scores at pre-treatment, using independent sample *t*-tests for continuous variables, and the chi-square test for the categorical variable. If significant, those would be co-varied in analysis.

Third, effects of MBSR on improvement in perceived stress and cognitive flexibility were examined using mixed-model repeated-measure ANOVAs using SPSS. A series of follow-up ANOVAs or *t*-tests were conducted following significant main effects and interactions.

Latent growth curve modelings (LGCMs) were conducted to explore the longitudinal trajectories of FFMQ total score and factor scores, and to investigate whether individuals or groups would differ in the initial levels and longitudinal changes in these scores. We estimated two latent factors (intercept and slope) across four waves (T1–T4). The intercept was defined by fixing the four parameters with a loading of 1.0, representing constant initial levels across four waves. The slope was fixed at loadings with 0, 3, 6, 8, representing the time spaces with T1. Group (0 = MBSR, 1 = waitlist) was incorporated as a covariate to test for the treatment effect on trajectories of mindfulness skills. LGCMs were administered using the Lavaan package (Rosseel, 2012) in R. Based on the criteria by Hu and Bentler (1999), CFI > 0.9, RMSEA < 0.08, and SRMR < 0.1 suggest a good fit of the model.

The mediation analyses were performed with Mplus version 5.2 (Muthén and Muthén, 2007). Adopting the method recommended by Baron and Kenny (1986), a mediation effect was determined by calculating the product of path coefficients constituting the indirect effect (e.g., path coefficient of the independent variable to mediator, and path coefficient of mediator to outcome variable) divided by bootstrapped standard error of this product. A bootstrap procedure was used to increase the statistical power (MacKinnon et al., 2002). We ran four separate mediation models to determine whether Non-reactivity at Week 3 or Week 6 and/or Non-judgment at Week 3 or Week 6, mediate the relationship between MBSR training and post-treatment cognitive flexibility. In each model, intervention group, which was transformed into dummy variable (0 = MBSR group, 1 = waitlist group), served as the independent variable. Post-treatment CFI scores served as the outcome variable. Thus, each model comprised of a path (“a” path coefficient) from group (MBSR or waitlist group) to mediator (Non-reactivity at Week 3 or Week 6, Non-judgment at Week 3 or Week 6), a path (“b” path coefficient) from mediator to outcome variable (post-intervention CFI score), and a direct path (“c′” path coefficient) from group to outcome variable controlling for mediator. The indirect effect of MBSR training on cognitive flexibility via the mediator is calculated by “a” multiplied by “b” (“ab” coefficient), the MODEL INDIRECT command was utilized in Mplus 5.2. A mediation effect was marked by a significant *ab* coefficient. In addition, goodness of fit parameters included comparative fit index (CFI), root-mean-square error of approximation (RMSEA), standardized root mean square residual (SRMR). According to the criteria by Hu and Bentler (1999), CFI > 0.9, RMSEA < 0.08, SRMR < 0.1 indicates good fit.

To examine the statistical power, we used Cohen’s *d* to calculate the effect size of *t*-tests. Cohen (1988) defined a small, medium, and large effect size as 0.2, 0.5, and 0.8. We also used partial η^2^ (Cohen’s *f*) to calculate the effect size of the main effects and interactions. A value of η^2^ ranging from 0.01 to 0.059 indicates a small, between 0.059 and 0.138 indicates a medium, and values ≥0.138 a large effect size (Cohen, 1988). We adopted the Monte Carlo method to calculate the power of mediation tests (Schoemann et al., 2017). The number of replication was set to 1000. For each replication, 200 times of random draws from the distribution of regression coefficients were used. As suggested by Cohen (1988), for the proportion of a variable explained by another variable, a small, medium, and large effect size was 0.01, 0.09, and 0.25, respectively.

## Results

### Demographical and Descriptive Data

The two groups were demographically matched and showed no significant difference in age (*t*_(52)_ = 0.24, *p* = 0.81, Cohen’s *d* = 0.07, 95% Confidence Interval (CI): −0.48 to 0.61), gender (*X ^2^_(52)_* = −0.26, *p = 0*.8), educational years (*t*_(52)_ = −0.2, *p* = 0.84, Cohen’s *d* = −0.05, 95% CI: −0.49 to 0.6), or per capita monthly income (*t*_(52)_ = 0.54, *p* = 0.6, Cohen’s *d* = 0.15, 95% CI: −0.49 to 0.6). There were no significant group differences in pre-treatment FFMQ (*t*_(52)_ = −0.82, *p* = 0.41, Cohen’s *d* = −0.22, 95% CI: −0.77 to 0.32), CFI (*t*_(52)_ = 1.36, *p* = 0.18, Cohen’s *d* = 0.37, 95% CI: −0.18 to 0.92) or CPSS (*t*_(52)_ = −0.67, *p* = 0.51, Cohen’s *d* = −0.18, 95% CI: −0.73 to 0.37) (see Table 1). Thus, no pre-treatment variables were co-varied in the follow-up analysis.

**TABLE 1:** Demographical and descriptive data for the MBSR and waiting-list group at baseline.

**Variables**	**MBSR (*n* = 26)**	**Waiting-list controls (*n* = 28)**	***t/X ^2^***	***p***	**Cohen’s *d***
Age	34.12 (7.63)	33.6 (8.24)	0.24	0.81	0.07
Gender (% Female)	69.23%	72.41%	–0.26	0.8	/
Educational years	17.82 (2.29)	17.93 (1.92)	–0.2	0.84	–0.05
Per capita monthly income (RMB)	21,653.94(30,479.14)	17,975.95(19,968.86)	0.54	0.6	0.15
FFMQ total score	107.88 (13.79)	110.95 (13.73)	–0.82	0.41	–0.22
Observing	24.65 (5.96)	25.67 (5.42)	–0.66	0.52	–0.18
Describing	23.12 (2.98)	23.25 (2.69)	–0.17	0.86	–0.05
Acting with awareness	17.73 (3.98)	18.63 (6.58)	–0.6	0.55	–0.16
Non-reactivity	20.35 (2.8)	19.62 (4.26)	0.74	0.46	0.2
Non-judgment	22.04 (5.42)	23.79 (6.13)	–1.12	0.27	–0.31
CFI	68.92 (4.46)	66.96 (5.87)	1.36	0.18	0.37
CPSS	43.96 (4.35)	44.74 (4.27)	–0.67	0.51	–0.18

*Gender was presented with Percentage, the other variables were presented with Mean (SD). MBSR = The Mindfulness Based Stress Reduction; FFMQ = The Five Facet Mindfulness Questionnaire, CFI = The Cognitive Flexibility Inventory, CPSS = The Chinese Perceived Stress Scale.*

In total, the drop-out rate was 20.37%. Six participants dropped out of the MBSR group (23.08%). Among them, one participant discontinued training at Week 3 because he was too busy, four participants did not attend the Week 6, for the reason of being too busy (*n* = 3) or taking care of children (*n* = 1). 1 participant did not complete the post-intervention, reporting being too busy. For the waitlist group, five participants dropped out (17.86%). Among them, one participant did not attend the Week 3 test, reason unknown. Four participants did not complete Week 6 test, for the reasons of being too busy (*n* = 3) or having a sprained foot (*n* = 1) (see Figure 1).

### Trajectory of Change in Mindfulness at Pre-treatment, Week 3, Week 6, and Post-treatment

The LGCMs analyses showed that only the Non-reactivity model had acceptable fit indices (CFI = 1, TLI = 1, RMSEA<0.001, SRMR = 0.067), whereas FFMQ total score and the other subscale models did not fit well (see Table 2). For Non-reactivity, the mean initial score was 21.75. The mean slope was 0.85, which was significantly different from zero (*p* < 0.001), suggesting a steady increase of Non-reactivity over time in the full sample. The value 0.85 can be interpreted as an average of 0.85 increase of Non-reactivity subscale score per unit of time. The variance of the intercept was 7.78 (*p* = 0.002), indicating significant individual variability of initial Non-reactivity score. The variance of slope and its covariance with intercept was not significant (*p* = 0.12 and *p* = 0.701, respectively). There was no significant group difference on the initial Non-reactivity subscale score (β = −1.36, *p* = 0.169). However, the factor Group (0 = MBSR, 1 = waitlist) showed a significant effect on the slope for Non-reactivity (β = −0.34, *p* = 0.019), indicating that the MBSR group increased faster than the waitlist group on Non-reactivity subscale score. Taken together, there was individual variability in Non-reactivity at the initial level, but the groups did not differ in the initial Non-reactivity score. On average, the slope grew over time. There was no individual difference in the growth rate. Group had a significant effect on the growth rate. The MBSR group increased at a faster speed than the waitlist control on the Non-reactivity score (see Table 3).

**TABLE 2:** Model fits based on the latent growth curve model of FFMQ scales.

**Variables**	**χ^2^**	**df**	**CFI**	**TLI**	**RMSEA**	**SRMR**
NR	5.16	7.00	1.00	1.02	0.001	0.07
FT	35.54***	7.00	0.72	0.61	0.30	0.40
NJ	35.13***	7.00	0.74	0.63	0.30	0.23
OB	18.12**	7.00	0.91	0.87	0.19	0.12
DE	38.69***	7.00	0.68	0.54	0.32	0.18
AW	74.86***	7.00	0.45	0.21	0.47	0.49

NR = the Non-reactivity subscale score, FT = the FFMQ total score, NJ = the Non-judgment subscale score, OB = the Observing subscale score, DE = the Describing subscale score, AW = the Acting with Awareness subscale score. **p < 0.01, ***p < 0.001.

**TABLE 3:** Parameter estimates based on latent growth curve model of FFMQ scales.

	**Estimate**	**SE**	***t***	***p***
NR				
Mean				
Intercepts	21.75	1.60	13.59	< 0.001***
Slope	0.85	0.23	3.67	< 0.001***
Variances				
NR1	3.56	1.79	1.99	0.047*
NR2	4.24	1.21	3.52	< 0.001***
NR3	4.06	1.34	3.03	0.002**
NR4	7.64	2.40	3.19	0.001**
Intercepts	7.78	2.52	3.09	0.002**
Slope	0.09	0.06	1.56	0.12
Covariance				
Intercept with slope	–0.12	0.31	–0.38	0.701
Regression				
Intercept on group	–1.36	0.99	–1.38	0.169
Slope on group	–0.34	0.14	–2.34	0.019*
FT				
Mean				
Intercepts	117.87	5.26	22.43	< 0.001***
Slope	5.17	0.79	6.54	< 0.001***
Variances				
FT1	422.34	87.08	4.85	< 0.001***
FT2	46.84	16.23	2.89	0.004**
FT3	56.08	20.30	2.76	0.006**
FT4	277.43	56.44	4.92	< 0.001***
Intercepts	–16.67	38.65	–0.43	0.666
Slope	–2.97	1.01	–2.93	0.003**
Covariance				
Intercept with slope	26.79	5.22	5.13	< 0.001***
Regression				
Intercept on group	–0.41	3.24	–0.13	0.9
slope on group	–2.02	0.49	–4.16	< 0.001***
NJ				
Mean				
Intercepts	24.04	1.48	16.23	< 0.001***
Slope	1.55	0.45	3.45	0.001**
Variances				
NJ1	37.09	7.90	4.69	< 0.001***
NJ2	24.76	5.04	4.91	< 0.001***
NJ3	25.34	5.49	4.62	< 0.001***
NJ4	–5.78	4.98	–1.16	0.246
Intercepts	–12.12	4.32	–2.81	0.005**
Slope	0.69	0.25	2.71	0.007**
Covariance				
Intercept with slope	0.33	0.75	0.43	0.665
Regression				
Intercept on group	0.32	0.91	0.35	0.725
Slope on group	–0.70	0.28	–2.52	0.012**
OB				
Mean				
Intercepts	25.29	2.82	8.96	< 0.001***
Slope	0.84	0.29	2.95	0.003**
Variances				
OB1	16.45	4.29	3.83	< 0.001***
OB2	3.51	1.60	2.20	0.028*
OB3	7.57	2.24	3.38	0.001**
OB4	16.37	4.29	3.82	< 0.001***
Intercepts	25.43	7.21	3.53	< 0.001***
Slope	–0.03	0.09	–0.28	0.783
Covariance				
Intercept with slope	–0.14	0.61	–0.22	0.823
Regression				
Intercept on group	–0.69	1.74	–0.40	0.693
Slope on group	–0.33	0.18	–1.90	0.057
DE				
Mean				
Intercepts	22.86	1.49	15.37	< 0.001***
Slope	0.98	0.21	4.64	< 0.001***
Variances				
DE1	8.66	2.63	3.29	0.001**
DE2	13.69	3.25	4.22	< 0.001***
DE3	10.40	2.88	3.61	< 0.001***
DE4	9.49	3.02	3.14	0.002**
Intercepts	2.15	2.69	0.80	0.424
Slope	–0.07	0.07	–0.95	0.342
Covariance				
Intercept with slope	1.35	0.35	3.81	< 0.001***
Regression				
Intercept on group	0.47	0.92	0.51	0.607
Slope on group	–0.13	0.13	–1.01	0.315
AW				
Mean				
Intercepts	19.76	0.93	21.21	< 0.001***
Slope	1.24	0.36	3.45	001**
Variances				
AW1	54.16	10.44	5.19	< 0.001***
AW2	16.21	3.25	4.99	< 0.001***
AW3	2.79	2.51	1.11	0.226
AW4	32.54	6.29	5.17	< 0.001***
Intercepts	–23.22	4.92	–4.72	< 0.001***
Slope	–0.28	0.17	–1.63	0.104
Covariance				
Intercept with slope	4.21	0.83	5.10	< 0.001***
Regression				
Intercept on group	0.80	0.57	1.40	0.163
Slope on group	–0.36	0.22	–1.60	0.109

NR = the Non-reactivity subscale score, FT = the FFMQ total score, NJ = the Non-judgment subscale score, OB = the Observing subscale score, DE = the Describing subscale score, AW = the Acting with Awareness subscale score. 1–4 = time points, *p< 0.05, **p< 0.01, ***p< 0.001.

### The Effect of MBSR on Perceived Stress and Cognitive Flexibility

For the CPSS scores, there was a significant main effect of Time (*F*_(1,52)_ = 313.78, *p* < 0.001, partial η^2^ = 0.86, 95% CI: 0.78–0.9), but the Group effect (*F*_(1)_ = 0.001, *p* = 0.99, partial η^2^ = 0.001, 95% CI: −0.55 to 0.55) and Time by Group interaction effects were not significant (*F*_(1,52)_ = 1.17, *p* = 0.29, partial η^2^ = 0.02, 95% CI: 0–0.15).

**FIGURE 2:**
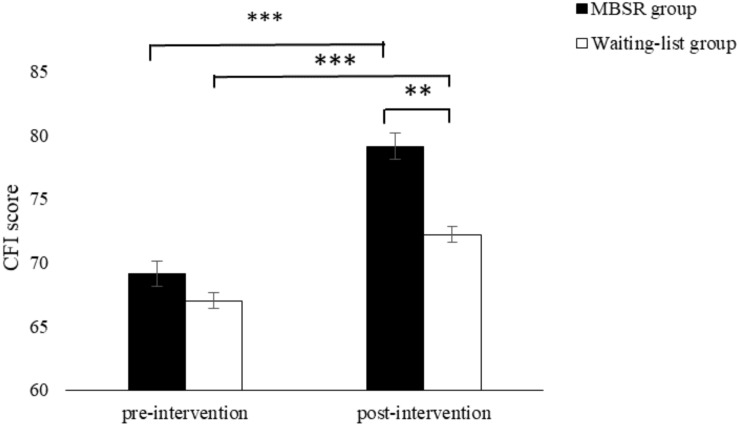
The Cognitive Flexibility Inventory scores as a function of Time and Group. Error bars represent standard error of the mean. **p < 0.01 and ***p < 0.001.

For the CFI scores, the Time effect (*F*_(1,52)_ = 59.86, *p* < 0.001, partial η^2^ = 0.53, 95% CI: 0–0.15), Group effect (*F*_(1)_ = 7.03, *p* = 0.01, partial η^2^ = 0.12, 95% CI: 1.25–2.57) and Time by Group interaction effect were significant (*F*_(1,52)_ = 4.27, *p* = 0.04, partial η^2^ = 0.08, 95% CI: 0–0.23). Follow-up *t*-tests showed that CFI scores increased significantly from pre- to post-treatment for both groups (*p*s < 0.001). However, the MBSR group scored higher than the waitlist control group at post-treatment (*p* = 0.003) (see Figure 2).

### The Mediating Effects of Non-reactivity and Non-judgment on the Relationship of MBSR and Cognitive Flexibility

The effect of MBSR on cognitive flexibility was not mediated by Non-reactivity at Week 3. The corresponding fit indices were reasonably good (*χ^2^*_(3)_ = 16.08, *p* < 0.001, CFI = 1, RMSEA < 0.001, SRMR < 0.001). However, the indirect effect was not significant (*ab* = −0.07, SE = 0.05, *p* = 0.14), despite a trend toward improved Non-reactivity at Week 3 via MBSR (*a* = −0.24, SE = 0.14, *p* = 0.08), and a statistically significant prediction on cognitive flexibility via Non-reactivity at Week 3 (*b* = 0.28, SE = 0.09, *p* = 0.002). The power of the indirect effect of Non-reactivity at Week 3 was 0.23.

**FIGURE 3:**
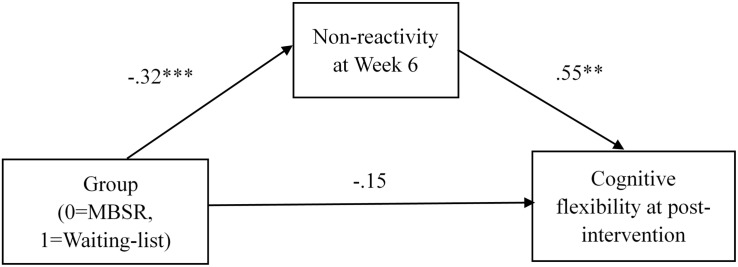
Path analysis illustrating the mediation effect of Non-reactivity at Week 6 on the relationship of MBSR training and cognitive flexibility. **p < 0.01 and ***p < 0.001.

There was a full mediation effect of Non-reactivity at Week 6. The model fit the data well (*χ^2^*_(3)_ = 39, *p* < 0.001, CFI = 1, RMSEA < 0.001, SRMR < 0.001). A significant intervention effect of MBSR on Non-reactivity at Week 6 was found (*a* = −0.32, SE = 0.11, *p* = 0.003), suggesting that MBSR improved Non-reactivity at Week 6. Furthermore, Non-reactivity at Week 6 positively predicted cognitive flexibility at post-treatment (*b* = 0.55, SE = 0.09, *p* < 0.001). The improvement of cognitive flexibility at post-treatment accounted by MBSR via Non-reactivity at Week 6 was significant (*ab* = −0.18, SE = 0.07, *p* = 0.008). Controlling for the mediating effect of Non-reactivity at Week 6, there was no significant association between group and cognitive flexibility at post-treatment (*c*′ = −0.15, SE = 0.08, *p* = 0.06), indicating a full mediation effect by Non-reactivity at Week 6 (see Figure 3). The mediating effect of Non-reactivity at Week 6 accounted for 53.94% of the total effect between group and cognitive flexibility. The power of the indirect effect of Non-reactivity at Week 6 was 0.69.

The effect of MBSR on cognitive flexibility was not mediated by Non-judgment at Week 3. Despite the good model fit (*χ^2^*_(3)_ = 24.62, *p* < 0.001, CFI = 1, RMSEA < 0.001, SRMR < 0.001), the indirect effect of Non-judgment at Week 3 was not significant (*ab* = −0.01, SE = 0.06, *p* = 0.82). The power of the indirect effect of Non-judgment at Week 3 was 0.04. The failure to find a significant mediating effect might be due to the disassociation between group and Non-judgment at Week 3 (*a* = −0.03, SE = 0.13, *p* = 0.82), indicating that there were no differentiated effects of MBSR training or waitlist assignment on Non-judgment at Week 3. There was a significant association between Non-judgment at Week 3 and cognitive flexibility at post-treatment (*b* = 0.45, SE = 0.07, *p* < 0.001).

Similar to Week 3, there was no mediating effect of Non-judgment at Week 6 on group and cognitive flexibility at post-treatment. The model fit the data well (*χ^2^*_(3)_ = 13.48, *p* < 0.001, CFI = 1, RMSEA < 0.001, SRMR < 0.001), but the indirect effect did not reach a significant level (*ab* = −0.09, SE = 0.08, *p* = 0.27). The power of the indirect effect of Non-judgment at Week 6 was 0.17. Despite the fact that Non-judgment at Week 6 predicted cognitive flexibility at post-treatment (*b* = 0.66, SE = 0.11, *p* < 0.001), improvements in Non-judgment at Week 6 could not be differentiated from waitlist group (*a* = −0.13, SE = 0.11, *p* = 0.24). Model fit indices please see Table 4.

**TABLE 4:** Model fit indices and standardized path coefficients for hypothesized mediation models.

**Model**	**χ^2^/df**	**CFI^2^**	**RMSEA**	**SRMR**	**Standardized path coefficients**	**IND %**
IV: Group	5.36	1	< 0.001	< 0.001	*a* = −0.24, SE = 0.14, *p* = 0.08	20.3
Mediator: Week 3 Non-reactivity					*b* = 0.28, SE = 0.09, *p* = 0.002**	
Outcome: Post-test CFI^1^						
					*c*′ = −0.26, SE = 0.09, *p* = 0.004**	
					*ab* = −0.07, SE = 0.05, *p* = 0.14	
IV: Group	13	1	< 0.001	< 0.001	*a* = −0.32, SE = 0.11, *p* = 0.003**	53.94
Mediator: Week 6 Non-reactivity						
					*b* = 0.55, SE = 0.09, *p* < 0.001**	
Outcome: Post-test CFI^1^						
					*c*′ = −0.15, SE = 0.08, *p* = 0.06	
					*ab* = −0.18, SE = 0.07, *p* = 0.008**	
IV: Group	8.2	1	< 0.001	< 0.001	*a* = −0.03, SE = 0.13, *p* = 0.82	4.24
Mediator: Week 3 Non-judgment					*b* = 0.45, SE = 0.07, *p* < 0.001**	
Outcome: Post-test CFI^1^						
					*c*′ = −0.32, SE = 0.08, *p* < 0.001***	
					*ab* = −0.01, SE = 0.06, *p* = 0.82	
IV: Group	4.49	1	< 0.001	< 0.001	*a* = −0.13, SE = 0.11, *p* = 0.24	26.97
Mediator: Week 6 Non-judgment					*b* = 0.66, SE = 0.11, *p* < 0.001***	
Outcome: Post-test CFI^1^						
					*c*′ = −0.24, SE = 0.09, *p* = 0.005**	
					*ab* = −0.09, SE = 0.08, *p* = 0.27	

IV = Independent variable, CFI^1^ = The Cognitive Flexibility Inventory, CFI^2^ = The Comparative fit index, RMSE *a* = Root-mean-square error of approximation, SRMR = Standardized root mean square residual, *a* = The path coefficient from IV to mediator, *b* = The path coefficient from mediator to outcome, *c*′ = The path of IV to outcome controlling for mediator, *ab* = a*b (the indirect effect), IND% = The percentage of the indirect effect on the total effect. **p < 0.01, ***p < 0.001.

## Discussion

The present work examined the efficacy of MBSR on cognitive flexibility in non-clinical stressed populations and the mediating effects of Non-reactivity and Non-judgment in explaining this effect. We put forward three hypotheses. First, we hypothesized that MBSR would be effective in improving cognitive flexibility, which was supported by the data. MBSR training had an immediate effect on cognitive flexibility with a medium effect size. Results also showed that compared with waitlist controls, MBSR training did not have incremental effect on stress reduction. Our second hypothesis was that Non-reactivity during intervention mediated the relationship between intervention group and cognitive flexibility. This hypothesis was partly supported. Non-reactivity at Week 3 did not mediate the association between Group and cognitive flexibility. However, Non-reactivity at Week 6 fully mediated the relationship between group and cognitive flexibility, which explained 53.94% of overall variances. Third, we hypothesized that Non-judgment during intervention mediated the relationship between group and cognitive flexibility, which was not supported by the data. Neither Non-judgment at Week 3 nor that at Week 6 mediated the association between MBSR training and cognitive flexibility.

Using a randomized controlled trial, we provided evidence that the MBSR program is effective in cultivating ability to generate alternative explanations. First, MBSR training lead to a significantly greater improvement in cognitive flexibility than waitlist controls. Second, the MBSR group achieved approximately 10 points increase in CFI from pre- to post-treatment, with a medium effect size. Our finding is consistent with another study that reported an 11-point increase in CFI for MBCT training group (Shapero et al., 2018). This finding is consistent with the general idea that MBI should improve the tendency to be mindful in everyday life, which might result in improvements in psychological outcomes, including responding adaptively to life events. Taken together, MBSR appears to be particularly effective in cultivating ability to perceive stressful life events as controllable and to form alternative explanations for stressful situations.

Unexpectedly, we did not replicate MBSR’s well-documented effect on psychological distress reduction (Chiesa and Serretti, 2009; Khoury et al., 2015; Ma et al., 2018), which is surprising. Four explanations are possible. First, demographical characteristics and baseline mindfulness might have confounded the treatment effect on the results. We conducted follow-up repeated-measure ANOVAs to include each of the potential covariates (e.g., age, gender, education, family income, initial level of FFMQ). The results showed that the Time by Group interaction was not significant (*p*s = 0.14–0.21), indicating that demographics and initial mindfulness level did not confound the training effect on stress. Second, participants in the MBSR group might have had a low basic stress level, which might lead to limited health benefits from the mindfulness training (Yu et al., 2019). We compared the baseline stress level with previous studies in stressed population without psychiatric disorders (Marcus et al., 2003; Chang et al., 2004; Yu et al., 2019). The abovementioned studies reported that the MBSR group had an average item score ranging from 1.8 to 2.7 points approximately at pre-treatment, whereas our sample in the MBSR group had an average item score of about 3 points, indicating a relative higher stress level, which did not support this explanation. Third, it is possible that participants in the waitlist group also experienced stress reduction over time. In fact, the waitlist controls in our study experienced substantially reduced stress from pre- to post-treatment (average item scores, pre-treatment: 3.2 points, post-treatment: 2.2 points). However, Yu et al. (2019) reported that waitlist controls perceived slightly higher stress from pre- to post-treatment (average item scores, pre-treatment: 1.8 points, post-treatment: 1.9 points). It is possible that the stress reduction effect for the waitlist controls in our study was driven by natural decay of stress or self-regulation, which warrants further investigations. Fourth, our assessment of stress (CPSS) emphasized the cognitive appraisal (e.g., uncontrollable, unpredictable, and unresolvable) of difficult situations, which did not include other aspects of stress responses (e.g., somatization). Therefore, it is possible that our assessment method was not sensitive enough to capture the severity of stress. These speculations warrant further investigations.

Non-reactivity at Week 6 during intervention had a full mediation effect on intervention group and cognitive flexibility. Our finding suggests that through 6 weeks of mindfulness practice, the Non-reactivity skill, which is about allowing experiences to come and go by themselves, without being attached to or changing them, was a successful and critical means to foster cognitive flexibility. These findings are in accordance with neuroimaging studies. For example, Creswell et al. (2007) found that individuals who showed less openness and acceptance to experiences, exhibited stronger activation in limbic systems when labeling negative thoughts and experiences. Whereas individuals who processed high openness and acceptance to experience exhibited stronger activation in prefrontal regions and inhibition of the limbic responses, suggesting successful inhibiting of habitual responses.

It is important to note that Non-reactivity at Week 3 did not mediate the relationship between intervention group and cognitive flexibility. This finding is not surprising. From path analysis statistics, failure to detect a mediation effect might be due to a disassociation between group and Non-reactivity at Week 3, which indicates the change of Non-reactivity was not attributed to the lack of effectiveness of MBSR. Tracing back to the 8-week MBSR program (Kabat-Zinn, 1990), the first three weeks introduced a small part of Non-reactivity skills. For example, participants were guided to experience bodily sensations, including exploring pain feelings and letting go of the reaction of changing the feeling of pain. In addition, participants were gradually guided to explore and experience emotional experiences, and to not take immediate action. But the most important exercises of Non-reactivity skills were introduced starting in Week 5. For instance, participants were guided to face the life stress, accept their own stress response, and temporarily not react so that they can get rid of the habitual reactions and eventually create a new way of coping. Therefore, for the MBSR group, the improvement of Non-reactivity from pre-treatment to Week 3 was far more subtle (mean at pre-treatment: 20.35, mean at Week 3: 21.56) than Week 6.

Our findings are consistent with the notions that Non-reactivity, cultivated by mindfulness, would alter the association between the perception and appraisal for the environmental stimuli (Lutz et al., 2015). Furthermore, this finding is convergent with growing evidence that non-reaction to emotion leads to beneficial psychological outcomes. For example, after a 3-month training, only Non-reactivity in FFMQ predicted greater reduction in mood symptoms in a present awareness mindfulness training group as compared to a progressive muscle relaxation training group (Gao et al., 2018). It has been suggested that adopting accepting-emotion strategies reduced negative affect (Campbell-Sills et al., 2006), and alleviated anxiousness and avoidance reactions (Levitt et al., 2004). In addition, prior research indicated that the improvement of the Non-reactivity facet from pre- to post-treatment mediated the effect of mindfulness training on decreasing depression symptoms from pre- to post-treatment in clinical samples (Heeren et al., 2015). These findings suggest that Non-reactivity may be a powerful mechanism of mindfulness. The underlying process might be that higher levels of Non-reactivity make it easier for people to disengage from established but unhelpful responses (Malinowski, 2013; Makowski et al., 2019), switch mental states adaptively, inhibit habitual responses, and thus have time to improve the ability to generate new appraisals and solutions to difficult situations. It is possible that the treatment targeted aspects directly linked to the content of the items included in the CFI. The CFI had a moderately positive correlation with the FFMQ at baseline (*r* = 0.35, *p* = 0.008), indicating a link between them. These speculations warrant further investigation. In summary, Non-reactivity is one important component of the effectiveness of MBSR training on cognitive flexibility.

For the longitudinal trajectories of FFMQ scales across four time points, we found the only the Non-reactivity scale fitted the latent curve model well. There was substantial individual variability in the initial score. All individuals increased on Non-reactivity at the same rate. However, the inclusion of group as a covariate resulted in a statistically significant growth rate. Specifically, the MBSR group increased at a faster speed than the waitlist controls.

Contrary to our hypothesis, Non-judgment during intervention did not mediate the effect of intervention group and cognitive flexibility. We make two speculations in explaining this finding. First, because some participants missed some assessment points, results might be biased. This is especially true for the waitlist control group, because those participants who persisted might have had greater interests in mindfulness than those who discontinued. Therefore, they might have been motivated to learn more about mindfulness from reading books or other materials, in which Non-judgment might be mentioned frequently. Over time, they would report a higher level Non-judgment skills, making the MBSR training effect of Non-judgment less notable. Second, improvements of Non-judgment at Week 3 and Week 6 were not explained by the efficacy of MBSR training. This speculation is supported by the fact that “a” path coefficients were not significant (for Non-judgment at Week 3: *a* = −0.03, SE = 0.13, *p* = 0.82; for Non-judgment at Week 6: *a* = −0.13, SE = 0.11, *p* = 0.24).

Several limitations should be considered. The present study had a small sample size, which might lower the statistical power. Our sample comprised of mostly women and highly educated participants, therefore conclusions should be taken with caution if generalizing to heterogeneous samples. We recruited participants without DSM-diagnosed mental illness, which may weaken the motivation of engagement with MBSR. In terms of practice duration, previous literatures showed that college students practiced 1.5 times/week (13 min/time) on average (Solhaug et al., 2019), whereas the mean practice durations in smokers were 20.89 min/day (Goldberg et al., 2014). Unfortunately, we did not collect the data of practice duration in this study to examine this issue. We used waitlist group (without any treatment) as controls, which could not explore the specific effect of mindfulness training. Measures were self-report instruments, which might be biased by retrospective memory and we did not assess actual life events. Future studies should consider measuring stressful life events, because cognitive flexibility may not only be trait-like stable but also context dependent (Schultz and Searleman, 2002). Furthermore, the missing outcome data might have biased the estimation of the treatment effect. Follow-up data were not collected, thus the maintenance effect remains in question. Future studies should use larger samples to test the replicability of this finding. Samples should include larger proportions of men and low-educated populations to test the generalization of the conclusion. Active controls such as psycho-education or relaxation training should be taken into account to test the specific effect of MBSR. Follow-up assessments would be needed to explore the maintenance effect, or the long-term benefits of improving cognitive flexibility. Finally, future studies are needed to examine other facets of mindfulness and their specific effects on stress and emotions (Carpenter et al., 2019).

Despite the abovementioned limitations, our findings exhibit sufficient statistical power and indicated that MBSR training is effective in improving flexible cognitions (perceived control and alternative explanations and solutions) confronting stressful life events, with a medium effect size. Furthermore, our findings suggest that Non-reactivity is the primary focus for MBSR training to increase cognitive flexibility. Our findings bridge knowledge gaps in prior studies by elucidating that Non-reactivity mediated the efficacy of mindfulness training on cognitive flexibility.

## Data Availability Statement

The datasets generated for this study are available on request to the corresponding author.

## Ethics Statement

This research involves human participants. All procedures performed in studies involving human participants were in accordance with the ethical standards of the research committee (the Association for Ethics and Human and Animal Protection in School of Psychological and Cognitive Sciences, Peking University, 2018-10-02) and with the 1964 Helsinki Declaration and its later amendments or comparable ethical standards. Informed consent was obtained from all individual participants included in the study.

## Author Contributions

YZ collected, analyzed, and interpreted the data, and wrote the drafts of the manuscript. PL collected the data. SH collaborated in the commenting on and editing the drafts of the manuscript. XL designed the study, taught part of the MBSR program, interpreted the findings, and commented critically on the drafts of the manuscript.

## Conflict of Interest

The authors declare that the research was conducted in the absence of any commercial or financial relationships that could be construed as a potential conflict of interest.
